# Evolution of cyclic di-GMP signalling on a short and long term time scale

**DOI:** 10.1099/mic.0.001354

**Published:** 2023-06-29

**Authors:** Ute Römling, Lian-Ying Cao, Feng-Wu Bai

**Affiliations:** ^1^​ Department of Microbiology, Tumor and Cell Biology, Biomedicum, Karolinska Institutet, Stockholm, Sweden; ^2^​ State Key Laboratory of Microbial Metabolism, School of Life Sciences and Biotechnology, Shanghai Jiao Tong University, Shanghai, PR China

**Keywords:** cyclic dinucleotides, biofilm, evolution, GGDEF, EAL, HD-GYP

## Abstract

Diversifying radiation of domain families within specific lineages of life indicates the importance of their functionality for the organisms. The foundation for the diversifying radiation of the cyclic di-GMP signalling network that occurred within the bacterial kingdom is most likely based in the outmost adaptability, flexibility and plasticity of the system. Integrative sensing of multiple diverse extra- and intracellular signals is made possible by the N-terminal sensory domains of the modular cyclic di-GMP turnover proteins, mutations in the protein scaffolds and subsequent signal reception by diverse receptors, which eventually rewires opposite host-associated as well as environmental life styles including parallel regulated target outputs. Natural, laboratory and microcosm derived microbial variants often with an altered multicellular biofilm behaviour as reading output demonstrated single amino acid substitutions to substantially alter catalytic activity including substrate specificity. Truncations and domain swapping of cyclic di-GMP signalling genes and horizontal gene transfer suggest rewiring of the network. Presence of cyclic di-GMP signalling genes on horizontally transferable elements in particular observed in extreme acidophilic bacteria indicates that cyclic di-GMP signalling and biofilm components are under selective pressure in these types of environments. On a short and long term evolutionary scale, within a species and in families within bacterial orders, respectively, the cyclic di-GMP signalling network can also rapidly disappear. To investigate variability of the cyclic di-GMP signalling system on various levels will give clues about evolutionary forces and discover novel physiological and metabolic pathways affected by this intriguing second messenger signalling system.

## Introduction

**Fig. 1.**
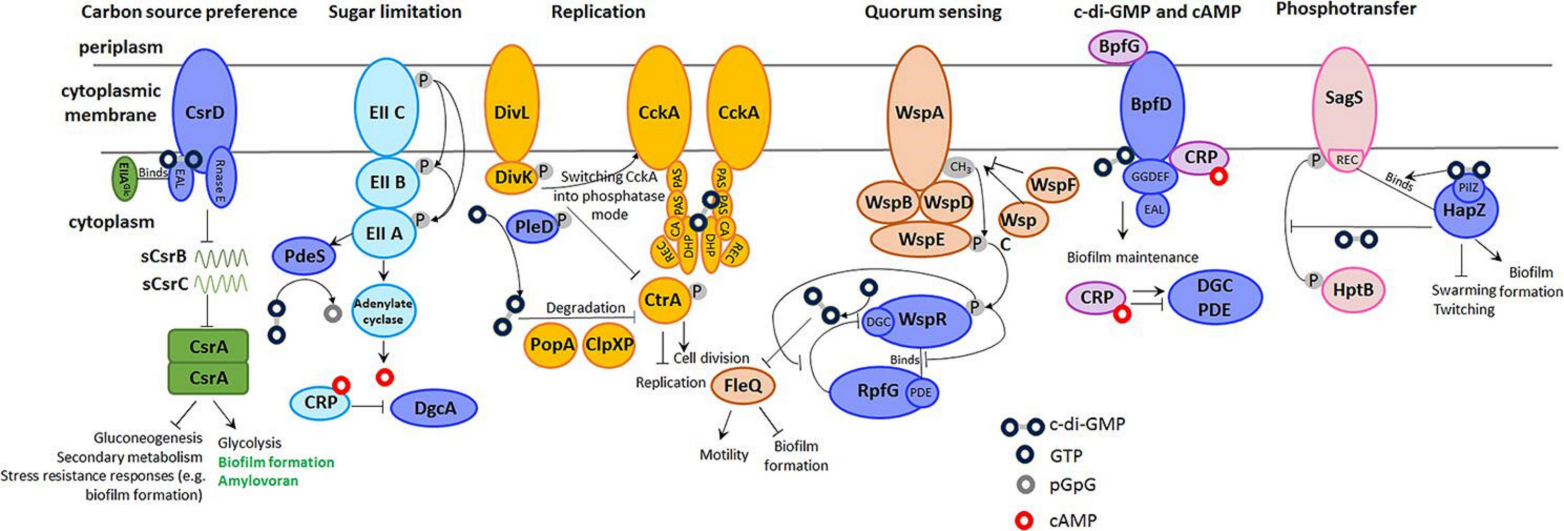
Crosstalk of cyclic di-GMP signalling with phosphotransfer, methyltransfer and second messenger signalling systems and small RNAs. The catalytically incompetent GAPES4-HAMP-dGGDEF-dEAL protein CsrD interacts with the small RNAs sCsrB and sCsrC to promote their degradation by RNase E in *E. coli* [162]. Binding of unphosphorylated EIIA^Glc^, a component of the phosphoenolpyruvate(PEP):carbohydrate phosphotransferase system (PTS), is required to activate degradation of sCsrB/C [163]. In the plant pathogen *Erwinia carotovora* the EAL domain of CsrC binds cyclic di-GMP to stimulate degradation of the small RNA sCsrB to promote production of the exopolysaccharide amylovoran and virulence [106]. As such, the target output is different in *E. coli* and *E. carotovora *, with biofilm formation promoted by CsrD activity in *E. carotovora* (in green). In the cholera pathogen *V. cholerae* C1552 the phosphodiesterase PdeS interacts with the phosphorylated PTS component EIIA^Glc^ to promote cyclic di-GMP degradation. Phosphorylation of the adenylate cyclase Cya promotes cyclic AMP synthesis, and subsequent cyclic AMP binds to the CRP transcriptional regulator which inhibits the transcription of the diguanylate cyclase CdgA. In *C. vibrioides *, cyclic di-GMP binds to the histidine kinase CckA to convert it into a phosphatase [164]. Cyclic di-GMP binding stabilizes ADP binding of a CckA tetramer which eventually leads to the degradation of the cell cycle regulator CtrA and inhibition of cell cycle progression. The Wsp chemosensory signal transduction pathway of *P. aeruginosa* and *P. fluorescens* stimulates autophorphorylation of the WspE histidine kinase which activates the response regulator WspR with a GGDEF output domain by phosphorylation [159, 165]. Cyclic di-GMP subsequently binds to the response regulator FleQ to inhibit motility and promote biofilm formation. In *L. enzymogenes *, the HD-GYP phosphodiesterase RfpG interacts and inhibits unphosphorylated WspR to act as an adaptor independent of its phosphodiesterase activity [166]. The transcriptional regulator CRP-cyclic AMP complex binds to the cyclic di-GMP receptor BpfD (a LapD homologue) in order to enhance biofilm formation by retaining the periplasmic protease BpfG in *Shewanella putrefaciens* [120]. In its original function, the CRP-cyclic AMP complex regulates the transcription of diguanylate cyclase and phosphodiesterase genes. In *P. aeruginosa *, the phosphotransfer from the histidine kinase SagS to the response regulator HptB is inhibited by the PilZ domain protein HapZ (PA2799) and further upon cyclic di-GMP binding in order to restrict swarming motility and promote biofilm formation [167].

Sensing and adaptation to different environmental conditions including nutrient and energy availability is essential for survival, persistence and proliferation of microorganisms. With these sensing mechanisms to coevolve with a shift or drift in the ecological niche and evolving speciation, the regulatory components such as signal transduction systems and targeted intergenic regions have been observed to be more variable than structural genes and open reading frames, respectively. Highly abundant and interconnected bacterial signalling systems based on methyltransfer, phosphotransfer and second messenger signalling (Fig. 1) have major fundamental physiological roles in the regulation of chemotaxis, remodelling of microbial physiology upon nutrient acquisition, adjustment of potassium and osmo-homeostasis, starvation, biofilm/motility, chronic/acute infection life style switch, differential expression of biofilm/virulence and microbial immune defence systems [1–4]. The diversifying radiation of the so-called GGDEF, EAL and HD-GYP protein domains that occurred within the bacterial kingdom led to the emergence of the cyclic di-GMP network as a ubiquitous second messenger signalling system. Although the evolutionary forces that selected preferentially cyclic di-GMP signalling among the various cyclic di-(and oligo-)nucleotides [5, 6] have not been defined, a multitude of cyclic di-GMP turnover proteins and diverse receptors integrate signals to regulate the multicellular predominantly sessile mode of growth (biofilm formation) versus motility equally as the acute/chronic infection life style switch on the single cell level upon response to intra- and extracellular signals. Being present in the deepest branching bacterial phyla and in over 75 % of all bacterial species throughout the phylogenetic tree, this signalling network provides levels of flexibility and variability that seemed to be incomprehensible among bacterial signal systems. The GGDEF, EAL and HD-GYP domains with virtually identical catalytic output as diguanylate cyclase and cyclic di-GMP specific phosphodiesterases, respectively, belong to the most abundant bacterial protein domains constituting protein superfamilies [7]. While GGDEF, EAL and HD-GYP catalytic domains can create local or global signals, the GGDEF-EAL combination can offer a tight spatial and functional covalent coupling of diguanylate cyclase and phosphodiesterase activity [8]. Alternatively, catalytically inactive domains can serve as receptors, sensory domains or solely through protein-protein interactions. The modular structure of the enzymes with conventionally one or multiple N-terminal signalling domains offers wide combinatorial options for the regulation of the catalytic output on the post-translational level. Post-translational modifications of cyclic di-GMP turnover proteins such as acetylation of the di-guanylate cyclase DgcZ causes decreased catalytic activity which can be significantly upregulated upon the action of the sirtuin two deacetylase CobB which is in a negative feedback loop inhibited by cyclic di-GMP [9]. Devoid of their N-terminal sensor domains, most of the GGDEF domains are monomeric with suboptimal catalytic activity, while dimerization and catalytic activity is stimulated by signal perception. Protein-protein interactions, allosteric feedback regulation and post-translational modifications contribute to regulation of activity and to maintain signal specificity [9–12]. Although the sequence identity can be below 20%, the outmost majority of cyclic di-GMP turnover proteins are readily recognized [12–14], while cyclic di-GMP binding sites in receptors are diverse and do not necessarily possess a common signature motif [8, 15]. While consecutive arginine residues such as the RxxxR motif and the RxxD motif can indicate a cyclic di-GMP binding site, the conformational flexibility of cyclic di-GMP and the requirement of only few amino acids to build up a binding site requires novel cyclic di-GMP binding sites to be experimentally identified. Few protein receptor binding sites possess an affinity in the nanomolar range equally as most cyclic di-nucleotide binding RNA aptamers, while most protein receptor binding site have a 100 to 1000-fold lower affinity in the µM range [15, 16]. In concurrence with its ancient phylogenetic origin and abundance [17], cyclic di-GMP signalling affects molecular mechanisms from modulating the binding properties of transcription factors to regulation of enzymatic activities and protein-protein interactions. Thereby, fundamental microbial physiological and metabolic processes can be affected which span from the oxidation of Mn^2+^, a process that is required for water clearance, the differential use of carbon sources and differential biofilm formation in host cells to the role of cyclic di-GMP as an extracellular signalling molecule [18–21]. Due to the sheer number of domains throughout the phylogenetic tree, the system is subject to substantial evolution. Variability can occur on different time and diversity scales from single nucleotide polymorphisms and loss of catalytic activity to gene truncation and horizontal gene transfer. We cover in this review exemplarly variability of the cyclic di-GMP and related cyclic di-nucleotide signalling systems.

## Point mutations in cyclic di-GMP turnover proteins

**Fig. 2.**
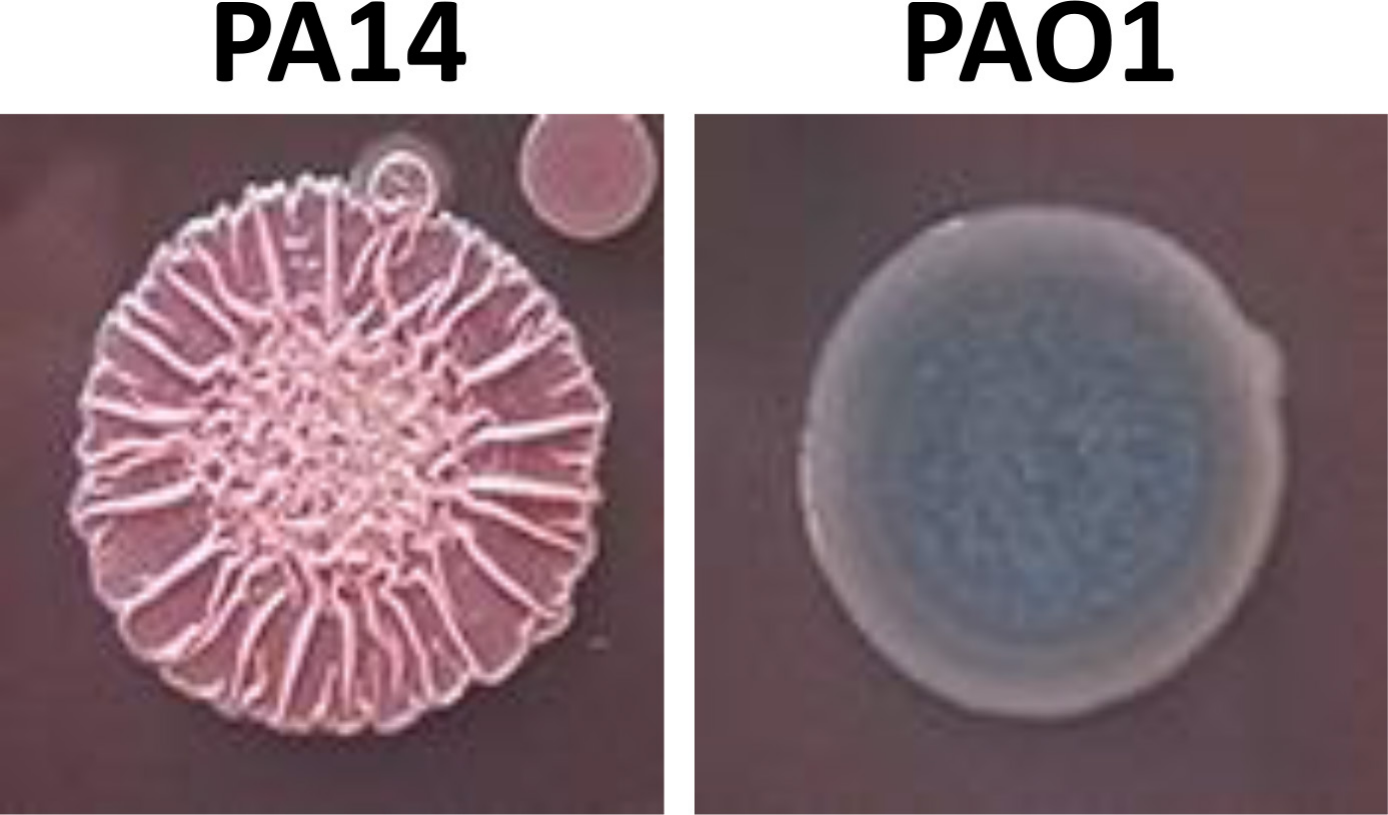
Example of an agar-grown biofilm colony morphotype versus non-biofilm colony type. Left: *P. aeruginosa* PA14 biofilm colony morphotype; right: *P. aeruginosa* PAO colony morphotype. Cells were grown at 28 °C for 2 days on 1 % tryptone agar. Picture taken in the author’s laboratory.

**Fig. 3.**
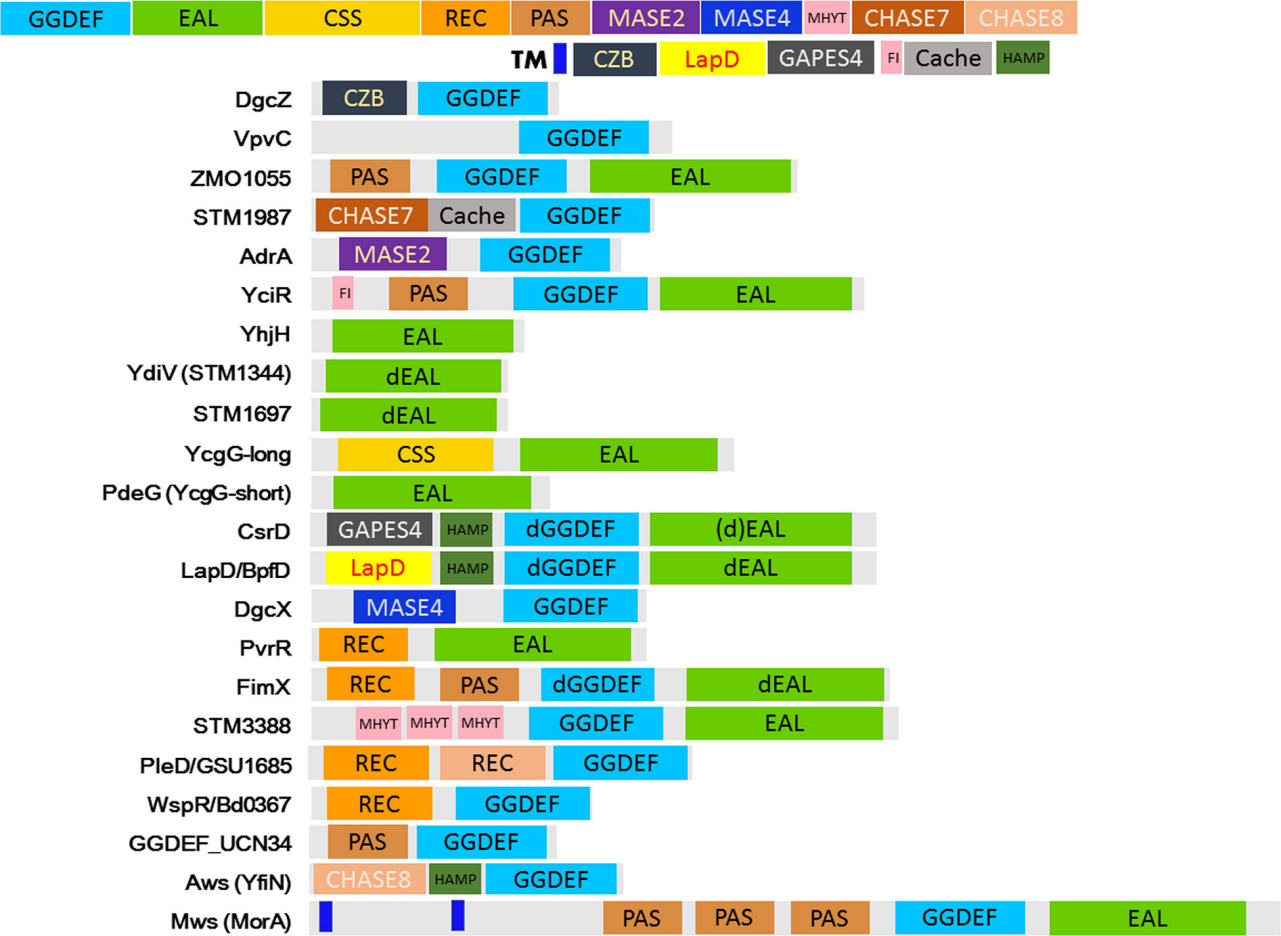
Domain structures of GGDEF and EAL domain proteins mentioned in this review.

Comparison of homologous cyclic di-GMP turnover proteins in different isolates within a species demonstrates that cyclic di-GMP turnover proteins can be present in different variants showing often only one amino acid substitution. However, as in most cases neither the biochemistry nor the phenotype of the microbial isolates can be connected to the alteration in the genome sequence (as such variability is mainly documented in databases), the impact of single amino acid substitutions on cyclic di-GMP turnover or signal perception is in most cases unclear. However, there are exceptions, in particular as biofilm formation with the expression of extracellular matrix components as a physiological output can often be readily displayed by a specific colony morphology which has been designated rdar (red, dry and rough), rugose or wrinkly on agar plates (Fig. 2; [22–26]). The impact of these observations, however, extends beyond laboratory investigations as, for example, *Vibrio cholerae* chlorine resistant rugose variants can be isolated from environmental sources [22, 27]. Spontaneous and induced alterations and phase variation in colony morphology from rugose/rdar/wrinkly to smooth or vice versa and other morphological switching events have led to the identification of single amino acid substitutions in cyclic di-GMP turnover proteins with up- or down-regulation of catalytic activities as consequence [28]. As such was the phase variation from a smooth to a rugose colony morphology caused by a single amino acid substitution (W240R) in the diguanylate cyclase VpvC (Fig. 3) of *V. cholerae* El Tor which also altered the susceptibility to phage infection [29]. Another example is the induced constitutive flocculation of *Zymomonas mobilis* ZM401 associated with the A526V substitution in the ZMO_1055 phosphodiesterase, a PAS-dGGDEF-EAL protein with degenerated GGDEF domain, to downregulate the catalytic activity of the EAL domain (Fig. 3; [30]). As these amino acid substitutions are located outside of the catalytic signature motifs, their impact on functionality is not immediately obvious. However, the investigation of the homologous proteins in closely related species indicates that the catalytic activities of ZMO_1055’s dGGDEF and EAL domain have been subject to evolution (Cao *et al*., manuscript in preparation). This scenario invites to investigate the correlation between single amino acid substitutions and catalytic activity in more detail.

The invasive *S. typhimurium* clone ST313 emerged in sub-Saharan Africa displays elevated virulence and impaired rdar biofilm formation. This alteration in the trade-off between virulence and persistence in the environment [31] is accompanied by multiple single nucleotide polymorphisms that target rdar biofilm formation (including mutations in the *csgD* promoter and leading to a truncation of the alkaline phosphatase superfamily member BcsG which is required for biosynthesis of the exopolysaccharide cellulose) [32, 33]. Furthermore, the single amino acid substitution T189R in the Cache sensory domain of the diguanylate cyclase STM1987 leads to diminished production of cellulose and enhanced virulence properties including increased replication in macrophages suggesting reduced diguanylate cyclase activity [32].

Oppositely, a transversion and the insertion of a single nucleotide in the promoter region of the major biofilm regulator *csgD* concomitantly led to the evolution from a regulated to a temperature and stress sigma factor RpoS independent rdar colony morphology and upregulation of the exopolysaccharide cellulose via the diguanylate cyclase AdrA in *Salmonella typhimurium* [23]. Single nucleotide polymorphisms that cause temperature independent rdar biofilm formation seem to occur under stress conditions as well as in clinical isolates to promote enhanced environmental survival and altered virulence [34–36].

Natural *Escherichia coli* isolates frequently display variability in biofilm formation. Specific alleles of the PAS-GGDEF-EAL domain protein YciR which atypically regulates rdar biofilm formation independent of its two catalytic activities in *E. coli* and *S. typhimurium* are associated with a temperature independent rdar morphotype [37]. In particular, two individual amino acid substitutions in the sensory and EAL domain of YciR created a protein that had a reduced ability to downregulate the rdar morphotype. The molecular mechanism(s) of inactivation of the suppressive activity of YciR which can be either by downregulation of YciR’s biofilm suppressing activity or YciR expression still need(s) to be determined.

However, also phenotypes other than biofilm formation are subject to cyclic di-GMP signalling mediated regulation. As a reversible mechanism, phase variation in the promoter region of a cyclic di-GMP specific phosphodiesterase has been observed to lead to a hypo- versus hyper-sporulation phenotype of *Clostridioides difficile* [38, 39].

## Alteration in substrate specificity of cyclic di-GMP turnover proteins

The majority of GGDEF domains exclusively synthesize the second messenger cyclic di-GMP as a product from two molecules of GTP. Thereby, the GG(D/E)EF motif tolerates amino acid substitutions such as (S/A)G(D/E)EF, substitution of the first glycine of the GGDEF motif by either alanine or serine, without the loss of catalytic activity [40]. However, a subset of GGDEF domains are promiscuous and either predominantly or as an additional major product synthesize cyclic AMP-GMP [41]. In the context of this specific GGDEF domain scaffold, the capability for cyclic AMP-GMP synthesis is determined by one amino acid substitution [41]. Changing the serine to aspartate, as found in the majority of diguanylate cyclases, 24 amino acids upstream of the GGDEF motif in the conserved GHL(I/V/A/F)GS motif of the GGDEF protein Bd0367 (and homologous GGDEF domain proteins) abolished cyclic AMP-GMP synthesis, but preserved synthesis of the product cyclic di-GMP and the minor product cyclic di-AMP *in vivo* and *in vitro* [42]. Of note, the aspartate at position three of the GGDEF motif is absolutely required for cyclic AMP-GMP synthase activity. Cyclic di-GMP and cyclic AMP-GMP have distinct functions in this bacterial predatory organism *Bdellovibrio bacteriovorus *, with cyclic AMP-GMP to promote gliding motility away from the bacterial prey, while cyclic di-GMP affects flagellar mediated swimming motility.

## Loss of catalytic activity of cyclic di-GMP turnover proteins

We assume that first GGDEF domains exclusively possessed catalytic activity as predicted for proteins found in members of the deepest branching phyla [17] and enzymatic inactivation of cyclic di-GMP turnover proteins to be a secondary event. An example of the subsequent loss of catalytic activity of cyclic di-GMP turnover proteins with development to be directly connected to biological relevance is provided in the evolution of the enteric pathogen *Yersinia pseudotuberculosis* to the flea-born pathogen *Yersinia pestis* [43, 44]. The subsequent inactivation of two functional phosphodiesterases by promoter mutations and pseudogene development accompany the speciation of the gastrointestinal *Y. enterocolitica* to flea-transmitted *Y. pestis* causing systemic infection. Dysfunctionality of these two enzymes enables robust biofilm formation of the *Y. pestis* pathogen in the flea gastrointestinal tract to subsequently promote efficient transmission to a human host.

Paralogous or xenologous proteins with evolved functionality can be encoded by one genome. YhjH is the only EAL only domain phosphodiesterase of *E. coli* and *Salmonella typhimurium* [45]. This cyclic di-GMP phosphodiesterase is dedicated to flagella-based swimming and swarming motility. YhjH regulates motility post-translational by adjusting motor speed and alternations in rotational direction leading to downregulation of flagellar motility [46]. Although with low sequence identity, two EAL only domain proteins, respectively, designated YdiV (STM1344) and STM1697, are still most similar to YhjH in the pathogenic strain *S. typhimurium* ATCC 14028 (Fig. 3). Those proteins do not possess catalytic activity nor do they bind cyclic di-GMP [47–49]. Both proteins, however, can interact with various transcription factors including FlhD_4_C_2_, the master flagellar regulon regulator and the ferric uptake regulator Fur. Homologous proteins that build up a subcluster of EAL domains with similar functionality in motility inhibition were identified in a number of gamma proteobacteria [50, 51]. Those EAL only proteins are an excellent example of accelerated signature motif versus domain evolution.

**Fig. 4.**
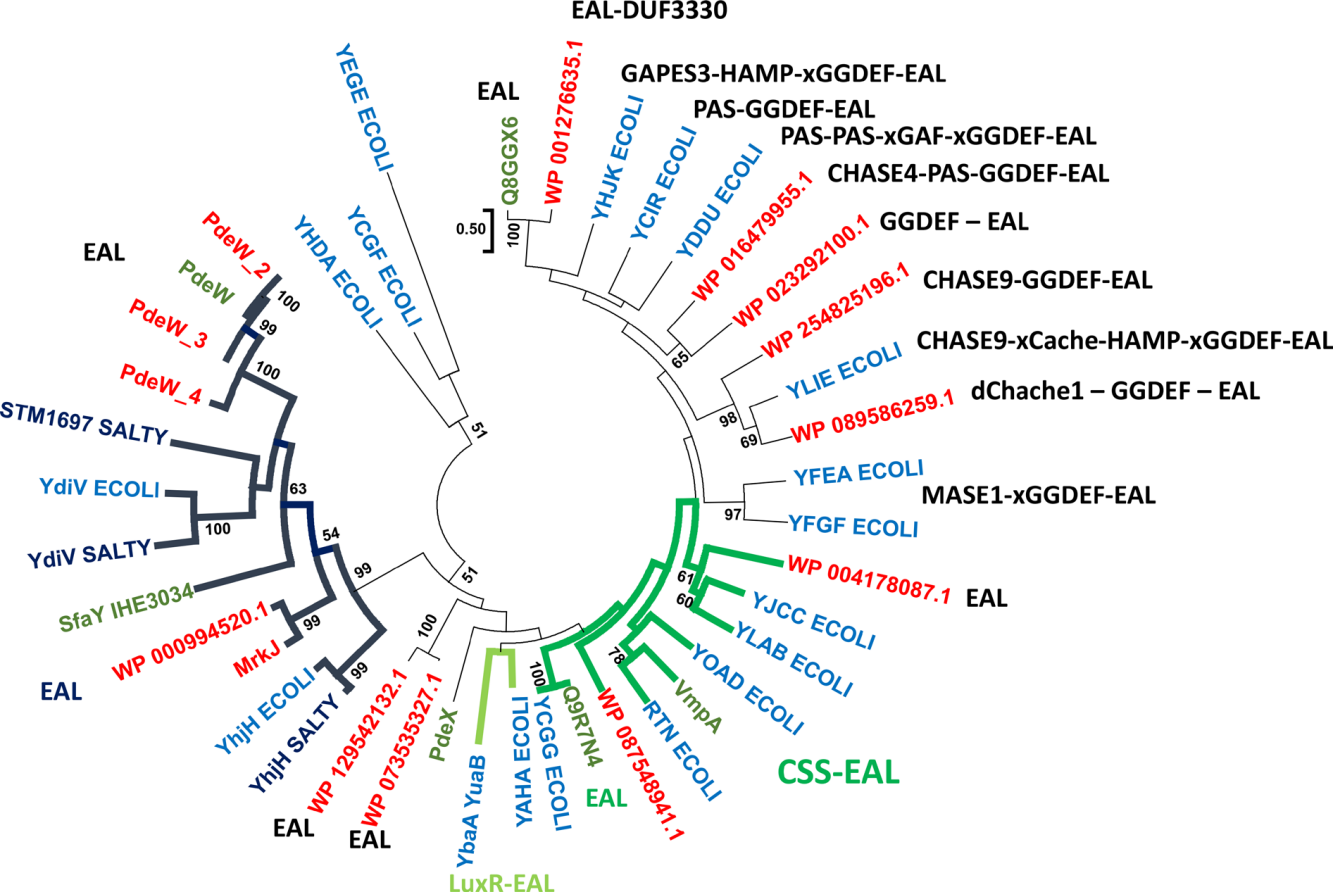
Phylogenetic relationship of core and accessory genome encoded EAL domains of *E. coli *. A wide variety of proteins with different domain structures have been subject to horizontal gene transfer into *E. coli* isolates. Core genome EAL phosphodiesterase domains in blue, previously identified horizontally transferred domains in light red and newly identified horizontally transferred domains in red. Reference *S. typhimurium* domains in dark blue. Note that the EAL domains predominantly but not exceptionally cluster with the domain structure of the proteins as indicated. EAL domains of proteins with identical domain structure forming a subgroup are indicated by thick dark blue lines (EAL only proteins), thick light green lines (LuxR-EAL proteins) and thick dark green lines (CSS-EAL). The EAL domains of the proteins were aligned in clustal x 2.1 and manually curated in GeneDoc [168]. The phylogenetic relationship was calculated in mega 7.0 or 10.0 using the Maximum Likelihood algorithm with phylogeny tested by 1000 bootstrap replicates indicating values over 50 % [169]. Size bar: 0.5 substitutions per 100 amino acids. Core genome proteins and previously identified horizontally transferred proteins taken from [60, 170, 171]. Horizontally transferred proteins were identified by blast search of *E. coli* genomes [172].

EAL only proteins of this subclass have also been found to be subject to horizontal transfer. *E. coli* isolates encode only YdiV, but plasmids with catalytically inactive EAL domain only proteins have been identified (Fig. 4). In *S. typhimurium *, the catalytically inactive EAL only proteins also regulate flagellar expression during systemic infection in the mouse host and inside host immune cells thus aiding innate and adaptive immune escape of bacterial cells, providing protection against reactive oxygen radicals and regulating flagellar motility upon nutrient starvation [52, 53].

In the fruit-rotting bacterium *Komagataeibacter xylinus *, three GGDEF-EAL domain diguanylate cyclases and cyclic di-GMP specific phosphodiesterases are highly homologous with their catalytic motifs conserved [54]. A highly degenerated loop six was identified to be responsible for the lack of catalytic activity of one of the EAL domains of the diguanylate cyclase [55]. Upon signal perception, loop six has been identified as being involved in cyclic di-GMP binding, divalent ion binding and domain dimerization. Downregulation of catalytic activity by a degenerated loop six can occur in approximately 50 % of EAL domains.

As another example of recent functional evolution homologues of the PAS-dGGDEF-EAL domain phosphodiesterase ZMO1055 of the biotechnologically relevant ethanol producer *Z. mobilis* possess a predicted catalytically functional GGDEF domain in *Spingomonas* spp. ([30]; Cao *et al*., manuscript in preparation). Identification of those examples of the evolution of vertical transmitted homologues (at the corresponding chromosomal location) provides the opportunity to investigate the evolutionary forces that drive the development towards inactivation of catalytic activity of GGDEF and EAL domains. A specific case is the GGDEF domain protein GdpS of *Staphylococcus aureus* and *Staphylococcus epidermidis *. Seemingly with all signature motifs for catalysis conserved, diguanylate cyclase activity is not involved in phenotypes regulated by GdpS including biosynthesis of the extracellular matrix component poly-N-acetyl-glucoseamine (PNAG) [56]. Evolved GGDEF domains can serve as cyclic di-GMP receptors (see below) or even act as sensory domains such as binding the substrate GTP to activate downstream EAL phosphodiesterase activity [57, 58]. As a more extreme deviation from its original enzymatic activity, a highly degenerated GGDEF domain connected to a DHH/DHHA1 phosphodiesterase domain which hydrolyses c-di-AMP and c-di-GMP has been shown to possess ATPase activity [59].

## Truncations and domain swapping in cyclic di-GMP turnover proteins

Evolution of cyclic di-GMP turnover proteins is constantly ongoing. Even in individual isolates within a species the cyclic di-GMP network can be highly variable. Single nucleotide polymorphisms, and gene truncations in cyclic di-GMP turnover proteins are found in different *E. coli* pathovars. A gene that is often truncated codes for the CSS-EAL domain protein YcgG ([37, 60, 61]; Cimdins-Ahne *et al*., manuscript in preparation). In *E. coli* phylogroup B2, *E. coli* ST131, neonatal meningitis causing *E. coli *, uropathogenic and commensal strains *ycgG* can be truncated. While the individual truncations, due to a deletion in the nucleotide sequence, are distinct, the truncations remove in any case almost precisely the N-terminal CSS signalling domain flanked by transmembrane helices leading to a cytoplasmic enzymatically functional protein. Intriguingly, this genetically fixed mutation is mirrored on the transcriptional and post-translational level by the location of two transcriptional start sites and by proteolysis, respectively [62]. Deletion of truncated *ycgG* in the pyelonephritis strain *E. coli* CFT073 displays differential expression of type one versus F1C fimbriae and a decrease in adherence to bladder epithelial cells [63]. While specific overall physiological and metabolic consequences of the truncation of *ycgG* still have to be unravelled, investigations in the neuroinvasive *E. coli* IHE3034 strain showed that a combination of reduced activity of the stress sigma factor RpoS and expression of truncated *ycgG* causes unique metabolic capabilities with acquisition of citrate-complexed Fe^3+^ by the citrate/succinate transporter CitT and upregulated Sfa fimbriae expression [60]. Deletion of truncated *ycgG* in the *E. coli* IHE3034 background leads to aerobic citrate fermentation in the presence of glucose.

Altered protein functionality with potentially altered physiology can also be created by domain swapping [64]. As such the deletion of a nucleotide sequence converted a cytoplasmic PAS-GGDEF domain protein to a membrane-associated diguanylate cyclase which is causative for elevated biofilm formation.

## Variability in gene content in cyclic di-GMP turnover proteins across species

Assessment of cyclic di-GMP turnover protein domains in members of the deepest branching bacterial phyla suggest early diversification of these domains [65]. Subsequently, additional diversifying radiation of these domain classes must have occurred with subsequently shrinkage of the domain number in selected bacterial families, genera or even species throughout the phylogenetic tree [66]. Thus, although the cyclic di-GMP mediated intelligence quotient, IQ (the density of the turnover domains per Mbp of genome), is roughly correlated with genome size within a bacterial phylum, it can vary widely, with the density of synthesizing and degrading proteins ranging from 0 to >36 cyclic di-GMP turnover proteins/Mbps (http://www-ncbi-nlm-nih-gov/Complete_Genomes/c-di-GMP.html). As an extreme, genomes of the same size can encode over 100 potential cyclic di-GMP turnover proteins or none. While aquatic microbial genera from the gamma-proteobacteria with its representatives such as *V. cholerae *, *Shewanella putrefaciens* and *Shewanella algae* consistently possess genomes with far above average density of cyclic di-GMP turnover proteins, the highest density is found in distinct Gram-negative and Gram-positive species such as sulphur-oxidizing *Sulfuricurvum kujiense *, iron-oxidizing *Gallionella capsiferriformans*, *Shewanella amazonensis* and acarbose producing *Actinoplanes* sp. (http://www-ncbi-nlm-nih-gov/Complete_Genomes/c-di-GMP.html). The catalytic domains of genome-encoded paralogous proteins are usually quite distinct with sequence identity of less than 35%, while close homologues are present in closely related species. Such cases can indicate ancient diversification into paralogues and vertical gene transfer on a short evolutionary time scale. Intriguingly, although few quantitative data are available, the cyclic di-GMP signalling network seems to evolve faster than any other signal transduction network [67, 68].

Upon alteration of life style, variability in gene content among species targets first the regulatory networks [68]. *Shigella* spp. evolved independently several times from *E. coli* by acquisition of an invasive intracellular life style. This life style change was accompanied by massive gene deterioration due to pseudogene development, IS element insertions and rearrangements of the chromosome which causes rewiring of physiology and metabolism. In particular, the cyclic di-GMP signalling network has been subject to evolution leaving only few cyclic di-GMP turnover proteins intact [68]. Despite this massive loss in functionality, the signalling network is still involved in the regulation of cell adherence, invasion and other phenotypes [69, 70].

Another example of a cyclic di-GMP turnover protein subject to evolution is the PAS-GGDEF-EAL domain protein YciR of *E. coli *. In contrast to other catalytically competent cyclic di-GMP turnover proteins, YciR negatively regulates rdar biofilm formation by cyclic di-GMP sensing and scaffold interaction with the transcriptional regulator MlrA [71]. Among the specific mutations associated with semi-constitutive rdar morphotype expression is a truncation of YciR precisely C-terminal of the GGDEF domain converting the truncated YciR into a diguanylate cyclase that activates the expression of the major biofilm regulator CsgD and the rdar morphotype [37].

## Variability in gene content among families – Morganellaceae

A current view is that bacteria that thrive in diverse environments maintain an extended cyclic di-GMP signalling system. Although cyclic di-GMP signalling seems to be essential for the fundamental process of cell cycle regulation [72], two major factors have been identified which force the cyclic di-GMP signalling system to rapidly disappear: reduction in genome size and host adaptation. A reduction in genome size eradicates the cyclic di-GMP signalling system faster than other signalling systems, although occasionally intracellular host adapted bacteria with small genomes such as *Rickettsia* and *Anaplasma pneumoniae* maintain a highly reduced cyclic di-GMP signalling system consisting of one diguanylate cyclase [73, 74]. Although present only sparcely in most Gram-negative bacteria, surprisingly, besides intracellular Gram-positive pathogens such as *Mycoplasma *, also Gram-negative pathogens such as *Chlamydia* spp*.* have maintained a cyclic di-AMP signalling system, but cyclic di-GMP signalling has not been maintained in this genus [75, 76]. Equally, already initial host adaptation can lead to a rapid deterioration of the cyclic di-GMP signalling system [68].

Remarkably, isolates of representative genera of the family Morganellaceae within the order Enterobacterales, but not representatives from other families such as Enterobacteriaceae, Erwiniaceae and Yersiniaceae, have nearly eradicated their cyclic di-GMP signalling system. *Proteus* spp. have, however, maintained one diguanylate cyclase and two cellulose biosynthesis systems predicted to be cyclic di-GMP dependent as judged from the presence of PilZ domains with conserved cyclic di-GMP binding motif at the C-terminal end of the cellulose synthase [66]. The genome size of environmental species such as *Providencia alcalifaciens* and *Photorhabdus luminescens *, an insect pathogen and mutualistic for nematodes, are in the range of 4.5 Mbp [77]; therefore no rational explanation for the degradation of the cyclic di-GMP signalling network is immediately available.

On the other hand, cyclic di-GMP signalling modules can be newly introduced into a genus. Such an example is the horizontal transfer of a cyclic di-GMP signalling module into *Streptococcus* spp. [66]. The module inserted into the serine tRNA locus between the core genome genes *trmB* and *rimP* involved in regulation of translation consists of a diguanylate cyclase, a Dpm1-GtrA hybrid protein, a cellulose synthase-like protein and an inner membrane protein with 16 predicted transmembrane helices. This diguanylate cyclase module has been found in probiotic species as well as animal pathogens of the salivarius, bovis, suis and pyogenes clade, while being excluded from the genomes of the human pathogens *Streptococcus pyogenes *, *Streptococcus pneumoniae* and *Streptococcus mutans *. Interestingly, *Streptococcus henryi* harbours two diguanylate cyclase one of them in a distinct genomic context and possesses a complex cyclic di-GMP phosphodiesterases ([66] and data not shown). A remarkable feature of streptococcal diguanylate cyclases is homology over the entire length of the protein with a conserved PAS-GGDEF domain structure, but surprisingly low sequence identity of the proteins among species of this genus.

## Cyclic di-GMP network components on mobile elements

One of the first EAL domain proteins identified is encoded by the *Tn*21 transposon [78, 79], although the precise function and biological role of this gene product still remains to be demonstrated. Also other transposons such as *Tn*501 can harbour cyclic di-GMP turnover proteins. In *V. cholerae* integrative conjugative elements encode cyclic di-GMP turnover proteins [80]. Few cyclic di-GMP elements have been identified on phages, such as a short GGDEF domain only protein on phage YuA and a cyclic di-GMP specific riboswitch on the PhiCD119 bacteriophage [81]. Other elements show direct evidence of horizontal gene transfer without being part of a mobile genetic element such as the diguanylate cyclase DgcX which is exclusively found in few *E. coli* strains including the outbreak strains *E. coli* ([82]; Cimdins-Ahne *et al*., manuscript in preparation). Other mobile genetic elements have been shown to encode cyclic di-GMP turnover proteins of wide physiological significance. Such disperses the cyclic di-GMP phosphodiesterase PvrR on the PAPI-1 genomic island biofilms of the pandemic *P. aeruginosa* PA14 clone [83]. A diguanylate cyclase and phosphodiesterase pair of the pathogenicity locus on a conjugative plasmid contributes to virulence of *Clostridium perfringens* in avian necrotic enteritis [84]. The transmissible locus of stress tolerance tLST represents a promiscuous genomic island found in major clones of gamma-proteobacterial pathogens [85]. Acquisition of diguanylate cyclases by the tLST island in *P. aeruginosa* and *S. typhimurium* includes a temperature sensing diguanylate cyclase that promotes robust biofilm formation of a *P. aeruginosa* isolate at high temperature [85–87]. A genomic island encoding chemotaxis and cyclic di-GMP turnover proteins is harboured by enteritidis causing *Shewanella algae* isolates suggesting its participation in the promotion of virulence [67]. In general, cyclic di-GMP turnover proteins are more than average found on plasmids as 6–10 % of plasmids harbour cyclic di-GMP turnover proteins. GGDEF domain proteins mostly on transmissible plasmids did not only enhance biofilm formation, but also the rate of conjugation [81] consistent with the observation that horizontal gene transfer is enhanced in biofilms.

Extreme acidophilic bacteria constitute diverse chemolithoautotrophic bacterial genera commonly with a pH optimum<=3 thriving in environments rich in iron and sulphur sources that serve energy gain. Extreme acidophilic bacteria contain integrative conjugative elements, IS-element rich regions and plasmids which are a sink for cyclic di-GMP elements, turnover proteins and receptors often also associated with biofilm genes [88, 89] indicating that in these environments biofilm formation is a transferable fitness factor.

## Variability of cyclic di-GMP binding in receptor families

Besides the cyclic di-GMP turnover proteins, cyclic di-GMP receptors are also subject to evolution with loss and gain of cyclic di-GMP binding sites ([90], Table 1). The most prominent example is PilZ, the first cyclic di-GMP receptor identified with the name-giving protein ironically not to bind cyclic di-GMP [91, 92]. PilZ is a small 116 aa long domain with highly variable sequence [93]. The cyclic di-GMP binding motif consisting of RXXXR and (D/N)XSXXG is located in the N-terminal part of the domain with ligand binding to cause substantial conformational changes. PilZ domains can be stand-alone proteins or occur in tandem involved in protein-protein interactions. More often though PilZ domains can also, by domain fusion, be part of a multidomain protein such as enzymes to affect their catalytic activity [93]. Non-canonical PilZ domains have still a role in cyclic di-GMP signalling as they can act as a scaffold in cyclic di-GMP signal transduction such as between the REC-PAS-dGGDEF-EAL cyclic di-GMP binding protein FimX and the PilB ATPase providing the energy for type IV pili polymerization and extrusion in *Xanthomonas axonopodis* pv. *citri* [94, 95]. While the C-terminal domain binds cyclic di-GMP, the N-terminal domain of PlzA, a tandem xPilZ-PilZ domain protein of the Lyme disease spirochete *Borrelia burgdorferi *, has been shown to be involved in DNA and RNA binding [96, 97]. In tandem xPilZ-PilZ domain proteins, such as in the flagellar motor break protein YcgR, the non-canonical xPilZ domain is often highly degraded giving rise to novel domain subclasses (Table 1; [98]).

**Table 1.** Cyclic di-GMP receptors and corresponding non-binding homologues

Cyclic di-GMP receptors or corresponding non-binding homologues	Representatives	Organism	Function	Cyclic di-GMP binding site	Reference
PilZ domain proteins					
xPilZ-PilZ	YcgR	* E. coli *	protein-protein interaction	RXXXR (D/N)xSxxG	[173]
xPilZ-PilZ	PlzA	*Borellia burgdorferi*	DNA/RNA binding of PilZ domain not binding cyclic di-GMP	RXXXR (D/N)xSxxG	[174]
effector-PilZ	BcsA	* E. coli *	cellulose synthase activity	RXXXR (D/N)xSxxG	[173]
xPilZ	PilZ	* Xanthomonas* spp.	interacts with cyclic di-GMP receptor FimX	no binding	[108]
PilZ	MapZ	* P. aeruginosa *	adaptor in chemosensory pathway	RXXXR (D/N)xSxxG	[175]
Transcription factors					
Clp/Crp transcriptional regulator	Clp (Xac0483, BCAM1349)	* Xanthomonas* spp., *Burkholderia *, *Stenotrophomonas maltophilia *, *Lysobacter enzymogenes *	regulator of biofilm formation and virulence	E RS/T TS/T/N, glutamate E99 required for cyclic di-GMP binding	[113–117, 119, 176]
	CRP	* E. coli *, *Pseudomonas putida *, *P. aeruginosa *, *V. cholerae *	catabolite repression (* E. coli *)	cAMP binding	
	CRP	* Mycobacterium tuberculosis *		cAMP binding	
	CRP	* Rhodospirillum centenum *	encystment	cGMP binding	[177]
CsgD subgroup LuxR type transcriptional regulator	VpsT	* V. cholerae *	biofilm activator	W[F/L/M][T/S]R	[111]
	CsgD	* S. typhimurium *, *E. coli *	biofilm activator	no binding	[112]
YajQ family	XC_3703/Smlt4090/PA4395/CdgL	* Xanthomonas campestris* pv *campestris*, *S. maltophilia *, *P. aeruginosa *, *L. enzymogenes *	interacts with LysR family transcription factor	n.d.*	[90]
	YajQ	* E. coli *, *Clostridium* sp., *Bacillus cereus *	interacts with transcription factor	ATP/GTP binding	[90]
P-loop NTPase lineage					
AAA +ATPase domain proteins					
NtrC type	FleQ/FlrA	* P. aeruginosa *, *V. cholerae *	sigma54 enhancer-binding transcription factor	LFRS RN ExxxR	[127, 178]
	FlrA	* V. cholerae *	sigma54 enhancer-binding transcription factor		[128]
	VpsR	* V. cholerae *	sigma54 enhancer-binding transcription factor, sigma70 associated	E R YxxxxxxRExxxQxxxxR	[129]
	Lon	* P. aeruginosa *; *V. cholerae *	proteolysis	n.d.*	[130]
PilB/GspE type	MshE (PilB, VC0405)	* V. cholerae *	polymerizing type IV pili ATPase	RLGxx(L/V/I) (L/V/I)xxG(L/V/I)(L/V/I)xxxxLxx xLxxQ	[15]
	MshE (GspE, PA14_29490)	* P. aeruginosa *	type II secretion system ATPase	RLGxx(L/V/I) (L/V/I)xxG(L/V/I)(L/V/I)xxxxLxx xLxxQ	[133]
	PilB	* Xanthomonas* spp., *P. aeruginosa *	polymerizing type IV pili ATPase	no binding	
PilT/VirD4 ATPases	PilT	* V. cholerae *	depolymerizing type IV pili ATPase	no binding	
RecA ATPases	RecA	* M. tuberculosis *, *Mycobacterium smegmatis *	recombinase	c-di-GMP and c-di-AMP binding; C-terminus	[135]
	RecA	* E. coli *	recombinase	c-di-GMP binding; C-terminus	[135]
Hybrid Hsp100 ATPases	ClpB2 (ClpV1)	* V. cholerae *	type VI secretion	c-di-GMP binding; n.d.	[134]
FliI/HrcN type ATPases	FliI/HrcN	* P. fluorescens *, *S. typhimurium *, *Sinorhizobium meliloti */* Pseudomonas syringae *	flagella/type three secretion system	R E R on different subunits	[134]
Degenerated cyclic di-GMP turnover proteins					
Degenerated EAL domains	FimX	*Xanthomonas, P. aeruginosa*	type IV pili biosynthesis, type III secretion system expression	EXLXR	[94]
	LapD/BpfD	*P. fluorescens, Bordetella bronchiseptica, Legionella pneumophila, Shewanella putrefaciens*	regulation of periplasmic LapG protease activity	EXLXR	[10, 120]
CsrD paralogues	CsrD	* Erwinia amylovora *	binding of CsrB/CsrC small RNAs for RNaseE degradation	EXLXR	[106]
	CsrD/MshH	*E. coli, S. typhimurium/V. cholerae*	binding of CsrB/CsrC small RNAs for RNaseE degradation	EXMXR, no binding	[107]
	PigX	* Serratia* sp.	CsrD homologue, apparent *in vivo* phosphodiesterase activity	catalytic activity	[105]
Degenerated GGDEF domain (examples)	PelD	* P. aeruginosa *	Pel polysaccharide biosynthesis	RxxD	[104]
	PopA	* C. vibrioides *	degradation of replication initiation inhibitor CtrA	RxxD	[179]
Receiver domain					
Pseudo-REC	ShkA	* C. vibrioides *	stimulation of phosphotransfer from histidine kinase to bEBP TacA	Y338	[136]
Pseudo-REC	Cle	* C. vibrioides *	interact with FliM upon cyclic di-GMP binding	[Y/F]XGPX[R/K]R	[137]
Conventional REC	PleD	* C. vibrioides *	phosphorylation of conserved aspartate 59 stimulates activity of output domain	no binding	[180]
Ribosomal components					
Elongation factor P	EF-P	*A. baumanii; Acinetobacter albensis*	facilitates relieve of stalling of proline-rich regions	K47, E69	[138, 139]
	EF-P	*E. coli, P. aeruginosa, and Burkholderia cenocepacia*	relieve of stalling of proline-rich regions	no binding	[138, 139]
Glycosyltransferase type 2					
	PgaC/PgaD complex	* E. coli *	PNAG (PGA/Ica) biosynthesis	R222A of PgaC	[140]
	PgaC/PgaD complex	* S. aureus *, *S. epidermidis *	PNAG biosynthesis	stimulation of PNAG synthesis does not require cyclic di-GMP	[56]

*n.d.=binding site not identified.

The PilZ domain has been the only cyclic di-GMP receptor predicted by bioinformatic approaches to bind cyclic di-GMP, while other cyclic di-GMP binding domains and their respective binding sites were experimentally identified [92]. Besides few amino acids to be required for binding of the molecule, the conformational flexibility of cyclic di-GMP allows multiple sterically distinct binding sites [99, 100].

**Fig. 5.**
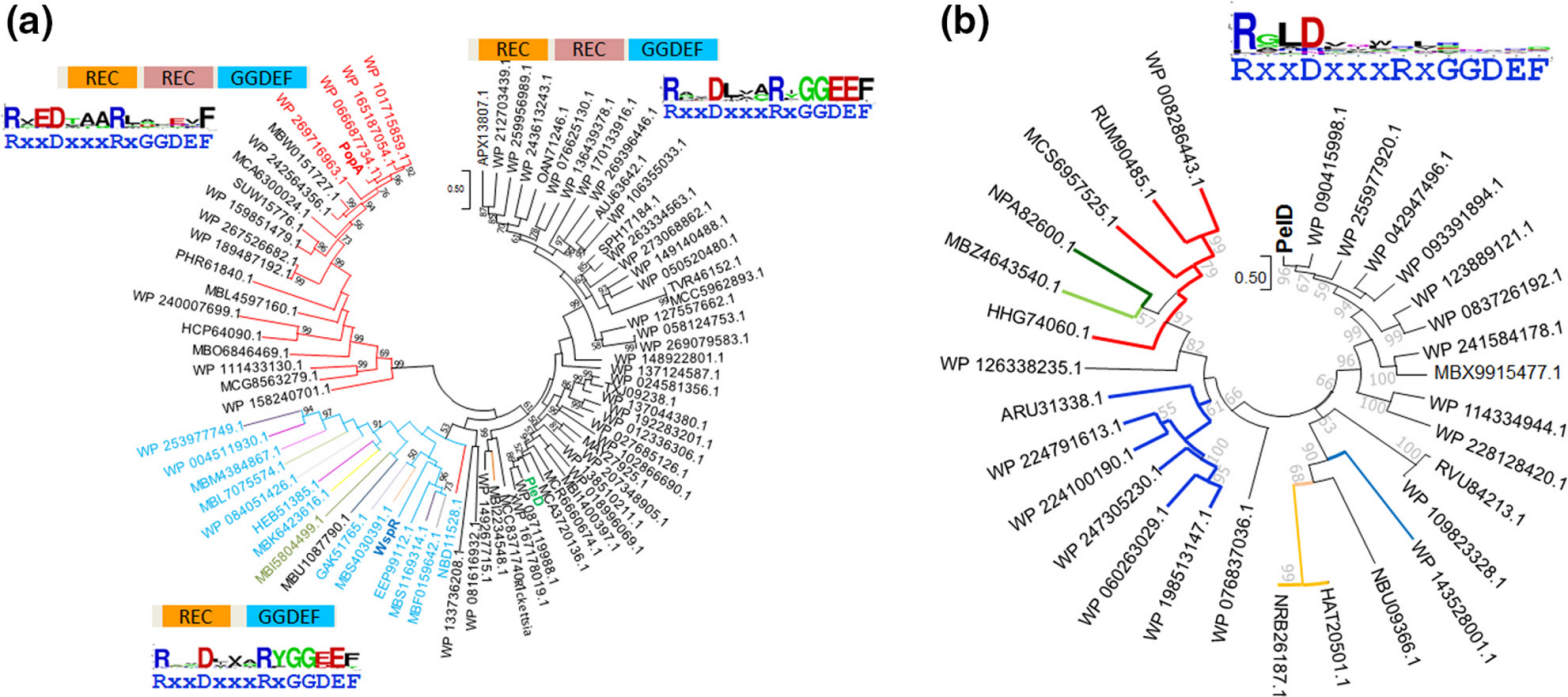
Phylogenetic trees demonstrating the different evolution of PopA and PelD receptors. (a) Phylogenetic relationship between PopA, PleD, reference WspR diguanylate cyclase and selected distant homologues. All protein homologues branching with PleD are predicted to possess catalytic activity. The REC-GGDEF diguanylate cyclase WspR and additional proteins that possess either no second REC domain (in light blue letters), a substitution of the second REC domain (in green letters) or another type of REC domain (in black letters) build up a separate branch in the tree. All protein homologues branching with PopA are predicted to be cyclic di-GMP receptors (as judged from the presence of the I-site) lacking catalytic activity (as indicated by the lack of conservation of the GGDEF motif). PopA receptors from *Caulobacter* species in red letters, PopA like receptors indicated with red branch lines. WspR class diguanylate cyclases in light blue. Proteins derived from other phyla or subphyla other than alpha proteobacteria within the Pseudomonadota are shown with a branch line other than grey, red or light blue. The sequence logo shows the RxxDxxxRxGGDEF motif site of displayed PopA, WspR and PleD proteins, respectively. (b) Phylogenetic relationship between PelD and selected distantly related homologues. All PelD homologues are predicted to be cyclic di-GMP receptors lacking catalytic activity according to the criteria mentioned under 5A. The sequence logo shows the RxxDxxxRxGGDEF motif site of displayed proteins. Proteins derived from other phyla or subphyla other than gamma proteobacteria within the Pseudomonadota are shown with a differentially coloured branch line. For example, red line=Aquificota. Homologues were identified by Blast search of the NCBI database and distantly related homologues with >90 % query coverage and a sequence identity of 23 % or higher were selected. Other experimental procedures as in Fig. 4.

GGDEF and EAL domains that have lost their catalytic activity have frequently evolved towards cyclic di-GMP receptors at different time scales. Many GGDEF diguanylate cyclases possess an allosteric inhibitory (I)-site, I-site, characterized by a RXXD motif and variable amino acid motifs elsewhere in the protein that restricts the catalytic activity [101]. Occasionally, RXXD independent binding sites have been identified in GGDEF domains. Upon loss of the catalytic activity, the dGGDEF domain can still maintain the I-site binding and serve as a cyclic di-GMP receptor [102]. Well-characterized receptors include the REC-REC-GGDEF domain protein PopA involved in cell cycle control in *Caulobacter vibrioides* by coordinating the degradation of the replication initiation inhibitor CtrA [103]. The membrane-integrated 3xTM-GAF-dGGDEF PelD of *Pseudomonas aeruginosa* is required for the post-translational activation of the PelD polysaccharide [104]. PopA equally as PleD seems to have evolved by accelerated diversifying radiation in alpha proteobacteria by acquisition of a second REC domain from a REC-GGDEF domain protein (Fig. 5a). PelD, on the other hand, seems to have evolved more anciently, as close homologues with predicted catalytic activity (by conservation of the GGDEF motif) cannot be readily identified even in deeply branching bacteria (Fig. 5b and data not shown).

Equally, a catalytically inactive EAL domain can still bind its substrate cyclic di-GMP with the ExLxR motif determinative for cyclic di-GMP binding (Table 1). The transition of the functionality of an EAL protein domain from enzymatic activity to a receptor or an alternative functionality can be fluid. In a *Serratia* sp., PigX, the paralogue of the catalytically incompetent GAPES4-HAMP-dGGDEF-dEAL protein CsrD of *E. coli* still possesses residual phosphodiesterase activity [105]. CsrD, on the other hand, has been observed to promote accessibility of the small RNAs CsrB/CsrC for cleavage by the RNase E in a cyclic di-GMP dependent and independent manner in the bacterial species *Erwinia amylovora* and *E. coli *, respectively [106, 107]. Indeed, a leucine to methionine amino acid substitution in the ExLxR motif is determination for cyclic di-GMP binding.

Other well-characterized GGDEF-EAL cyclic di-GMP binding proteins include the REC-PAS-dGGDEF-dEAL domain protein FimX family. This cytoplasmic receptor family is involved in polar biogenesis of type IV pili by activation of the ATPase PilB upon cyclic di-GMP binding via a PilB-PilZ-FimX complex and type III secretion system expression in *Xanthomonas* spp. [108, 109]. Another well characterized phylogenetically widespread system within Pseudomonadota is the cyclic di-GMP receptor LapD which regulates, upon cyclic di-GMP binding, the differential functional production of at least two large surface adhesins including LapA in *Pseudomonas fluorescens* by inside-out signalling via periplasmic capture of the LapG protease [10, 110]. In contrast to the above described dGGDEF domain derived cyclic di-GMP receptors PopA and PelD, the development of, for example, the CsrD protein from a catalytically competent enzyme towards an EAL-based cyclic di-GMP receptor and into a cyclic di-GMP non-binding protein seems to be more recent as homologous proteins with an identical domain structure and catalytically functional motifs can be identified or have even been experimentally demonstrated in phylogenetically closely related species (Table 1). Intriguing is the question, how, why and by which mechanisms cyclic di-GMP turnover proteins have developed into non-catalytic and/or even non-binding entities.

A wide variety of members of families of transcriptional regulators bind cyclic di-GMP differentially. Among those cyclic di-GMP binding proteins are central biofilm activators such as the subgroup of CsgD-like non-canonical response regulators of the FixJ/LuxR family. In *V. cholerae *, the response regulator VpsT binds cyclic di-GMP to activate production of the VPS exopolysaccharide while motility is inhibited [111]. Surprisingly though, CsgD, the central activator of rdar biofilm formation of *S. typhimurium* and *E. coli* does not bind cyclic di-GMP and activates transcription of target genes in its non-phosphorylated state [112].

**Fig. 6.**
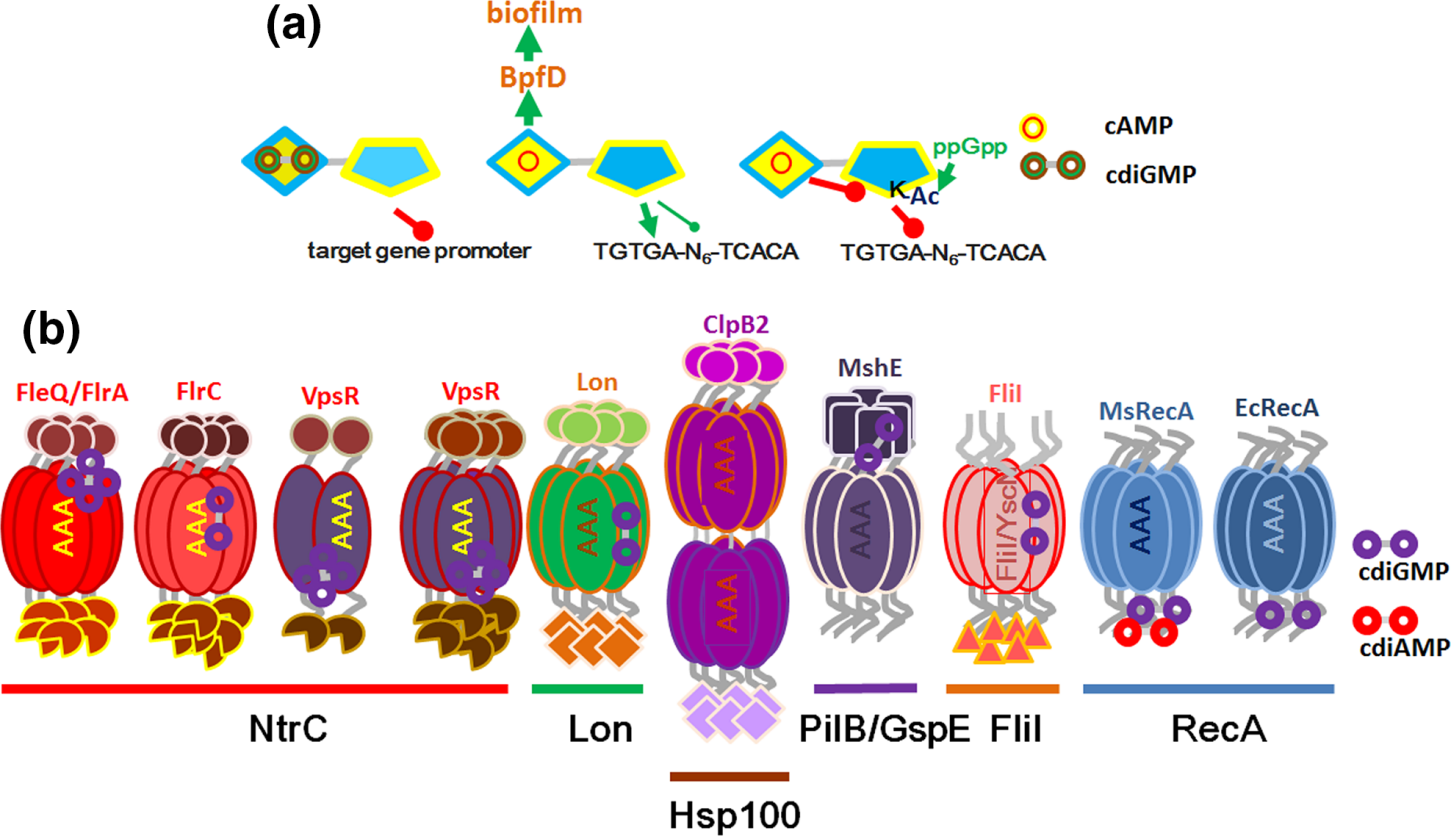
Domain structure of selected cyclic di-GMP receptors with variable binding sites. (a) Divergent functionality of cyclic di-GMP binding Clp and cyclic AMP binding CRP transcriptional regulators. Clp proteins bind cyclic di-GMP via their N-terminal cyclic nucleotide binding domain which inhibits binding to target promoters to either stimulate or restrict transcription. In contrast, cyclic AMP binding to the sensory domain of CRP proteins promotes binding to the target promoter region to stimulate transcription in most cases. Cyclic di-GMP turnover proteins are among the genes targeted by cAMP-CRP. In a second functionality cAMP-CRP can bind to BpfD, a LapD type cyclic di-GMP receptor to promote biofilm formation via inhibition of the periplasmic protease BpfG. Cyclic AMP binding inhibits acetylation of a lysine residue in the DNA binding domain, while ppGpp promotes acetylation. Green activating and inhibiting arrows indicate the effect on gene transcription, red inhibiting arrows indicate lack of binding to target promoters. (**b)** P-loop NTPase domains as cyclic di-GMP receptors. Representatives of different classes of P-loop ATPases bind cyclic di-GMP and cyclic di-AMP with different binding modes. VpsR, an atypical NtrC family protein dimerizes upon cyclic di-GMP binding and forms non-functional oligomers at higher cyclic di-GMP concentration. Further explanation see text.

The CRP/FNR family of transcriptional regulators was initially characterized to bind cyclic AMP with the active Crp-cyclic AMP complex in *E. coli* as a prototype (Fig. 6a). Subsequent analysis showed that a subgroup of the CRP/FNR family characterized by the conserved signature glutamate E99 demonstrated cyclic di-GMP binding instead [113–119]. Binding of cyclic di-GMP represses the DNA binding activity of these Clp (CRP-like proteins). Cyclic AMP signalling is otherwise cross-talking with cyclic di-GMP signalling via CRP not only via transcription of cyclic di-GMP turnover proteins, but unconventionally also by binding to the BpfD effector, a homologue of the LapD effector of *P. fluorescens *, to enhance biofilm formation of *Shewanella putrefaciens* [120] and, indirectly, by acetylation of CRP which is inhibited by cAMP binding and promoted by ppGpp [121, 122].

The YajQ family is a large cytoplasmic family of potentially nucleic acid interacting small nucleotide binding proteins. In this family, the 161 aa long YajQ of the plant pathogen *Xanthomonas campestris* has been identified as a cyclic di-GMP binding protein [123]. YajQ’s interaction with a HTH-LysR type regulator leads to the binding to target promoters involved in virulence and biofilm formation. Upon cyclic di-GMP binding, the promoter binding activity is abolished. In the fungal predator *Lysobacter enzymogenes* OH11A, a YajQ homologue (CdgL) interacts with the transcription factor LysR whereby cyclic di-GMP binding weakens the interaction leading to decreased expression of the heat-stable antifungal factor HSAF biosynthesis operon [124]. Cyclic di-GMP binding is, however, not a general feature of members of the YajQ family with *E. coli *, *Clostridium* sp. and *Bacillus cereus* representatives not binding cyclic di-GMP, but ATP or GTP.

The P-loop NTPase is an ancient monophyletic domain lineage diversified into domain (super)families with diverse functionality of its members. Representatives of different families of P-loop ATPases have been shown to bind cyclic di-GMP and cyclic di-AMP by different mechanisms and with different biochemical outcomes (Fig. 6b, Table 1). Within the P-loop ATPase lineage, the ASCE group of ATPase domains as part of multidomain proteins constitutes a diverse domain class involved in diverse processes such as transcriptional activation, protein unfolding and DNA replication [125, 126]. Already among the family of bacterial enhancer binding proteins (bEBPs) with a REC-AAA +ATPase-Fis-like helix-turn-helix DNA binding domain structure there is a diversity of binding modes and biochemical consequences. The bEBP sigma 54 dependent transcription factor FleQ of *P. aeruginosa* binds cyclic di-GMP outside of the ATPase catalytic centre of the conventionally hexameric AAA +domain (Fig. 6; [127]). The cyclic di-GMP binding heptameric bEBP FlrC acts downstream of the FleQ homologue FlrA in the flagellar regulon cascade of *V. cholerae* [128]. Upon cyclic di-GMP binding, those proteins are disassembled with ATPase activity repressed. In contrast, the atypical bEBP VpsR assembles into a functional dimer upon cyclic di-GMP binding concomitant with stimulation of the ATPase activity of the AAA +domain [129]. The AAA+ ATPase domain protease Lon is inhibited by cyclic di-GMP, but the binding site for the cyclic di-nucleotide has not yet been identified [130, 131]. On the other hand binds the MshE type P-loop ATPase within the N-terminal T2SSE_N domain cyclic di-GMP with high affinity mainly by hydrophobic interactions [132, 133]. Such MshE type ATPases (also designated GspE/PulE/PilB type) include GspE ATPases involved in type II protein secretion in *P. aeruginosa* and PilB ATPase involved in type IV pili assembly in *V. cholerae *. In contrast, PilB ATPases of *Xanthomonas* spp. do not bind cyclic di-GMP, but interact with a PilZ and cyclic di-GMP binding FimX (REC-PAS-dGGDEF-dEAL) protein to promote type VI pilus biogenesis [108]. FliI/YscN type rotary export ATPases are another P-loop family which bind cyclic di-GMP at the interface between two subunits of the hexameric protein [134]. While the Hsp100 ClpB like ATPase ClpB2 (ClpV1) regulating type IV secretion binds cyclic di-GMP, identification of the binding motif is still elusive [134]. The RecA-like ATPase domain of the RecA recombinase of *Mycobacterium tuberculosis* MtRecA and *Mycobacterium smegmatis* MsRecA binds cyclic di-AMP and cyclic di-GMP at the genus-specific C-terminal motif, while *E. coli* EcRecA binds exclusively cyclic di-GMP consistent with the absence of a cyclic di-AMP network in this species [135].

In bacterial two-component systems, the receiver domain REC is conventionally the N-terminal sensory domain of response regulators which is phosphorylated at the conserved aspartate 59 by the cognate sensory histidine kinase. In *C. vibrioides *, variants of the receiver domain were recently shown to bind cyclic di-GMP [136, 137]. Those pseudo-receiver domain occur in proteins with diverse functionalities with communalities of cyclic di-GMP binding pseudo-receiver domains still to be defined.

The elongation factor P of *Acinetobacter baumanii* is another protein receptor with narrow genus specific cyclic di-GMP binding capability [138, 139]. Binding of cyclic di-GMP by elongation factor P relieves the stalling of proline-rich regions during translation thus promoting biofilm formation and virulence.

Furthermore, the catalytic activity of the PgaC glycosyltransferase type 2-PgaD complex constituting the synthase for the biofilm exopolysaccharide PNAG is delicately regulated by cyclic di-GMP binding in the Gram-negative bacterium *E. coli *, but not in Gram-positive *S. epidermidis* and *S. aureus* [56, 140].

Last, but not least, cyclic di-GMP can also be recognized by RNA aptamers with affinity in the nM range [16]. Guanosine at position 20 of the cyclic di-GMP-I riboswitch in conjunction with additional nucleotides in the aptamer backbone has been found determinative for preferential binding of cyclic di-GMP against cyclic AMP-GMP [141].

## Conservation of cyclic di-GMP components throughout the phylogenetic tree

Although GGDEF and EAL domains can be subject to rapid evolution, on the other hand cyclic di-GMP turnover proteins can also be highly conserved throughout the phylogenetic tree. One or multiple amino acid substitutions can occur in homologous cyclic di-GMP turnover proteins from different isolates of a species, and highly conserved orthologous/paralogous proteins with a similar domain architecture can be found in different, often even distantly related species. Those orthologous/paralogous proteins can have adapted a novel functionality with respect to the regulation of a distinct target output in the species, but can otherwise also be surprisingly conserved in functionality. A prominent example is the STM3388 MHYT-MHYT-GGDEF-EAL domain protein of *S. typhimurium* that differentially regulates the central biofilm regulator CsgD during the growth phase [142, 143]. A paralogous gene is present in the environmental pathogen *P. aeruginosa* in order to sense nitric oxide, nitrate and to activate biosynthesis of the exopolysaccharide alginate [144].

## Variability in model systems *Pseudomonas fluorescens *, *Pseudomonas aeruginosa *, *Burkholderia cepacia* and *Xanthomonas retroflexus*

*In vitro* evolutionary biofilm model systems have been developed in order to mimic adaptation of bacterial species to different habitats including mimicking host conditions providing the basis for chronic infections. Such model systems of adaptive evolution have been proven to be a treasure source to detect mutations in the cyclic di-GMP signalling system and to unravel previously unknown regulatory mechanisms. Mutants of *P. fluorescens* SBW25 emerge in a heterologous microcosm, a static culture system, with preferential occupation of distinct niches. As an efficient coloniser of the air-liquid interface consistently a so-called ‘wrinkly spreader’ colony morphotype evolves showing a distinct upregulated biofilm phenotype on agar plates. As the molecular basis of enhanced biofilm formation, mutations that lead to upregulation of the cyclic di-GMP signalling system with subsequent overproduction of an acetylated cellulose exopolysaccharide have been identified. Predominantly three cyclic di-GMP signalling pathways; a chemotaxis-like system with di-guanylate cyclase output of the response regulator WspR; the Aws (YfiN) di-guanylate cyclase gene cluster and the diguanylate cyclase Mws (MorA) were identified to be targeted [145–147]. Mutations that arise upon deletion of these three major c-di-GMP synthesizing pathways still occur targeting mainly diguanylate cyclase activity as loss-of-function mutations in a negative regulator, amino acid substitutions in di-guanylate cyclases and gene fusions in different diguanylate cyclases, promoter mutations and combinatorial mutations in cyclic di-GMP turnover proteins [148]. Upon deletion of the cellulose biosynthesis operon, alternative pathways of air-liquid interface colonization are production of the exopolysaccharide poly-N-acetyl-glucosamine and the *nlpD* lipoprotein gene product that activates the amidase AmiC to display a cell chaining phenotype [149].

Intriguingly, the Wsp chemotactic signalling pathway and the YfiBNR system are also major targets of mutational events leading to the development of small colony variants of *P. aeruginosa* upon colonization of the cystic fibrosis lung [150, 151] and in rhizosphere-colonizing *Pseudomonas chlororaphis* [152]. Similarly, mutations in the Wsp chemotactic pathway arise readily upon chronic infection in a full-thickness burn wound model [153], the challenge of *P. aeruginosa* with sub-lethal concentrations of hydrogen peroxide, the subjection of *Burkholderia cenocepacia* to a heterogeneous microcosm [154], other infections where hyper biofilm producers with elevated cyclic di-GMP levels are observed [155] and also upon biofilm formation of oceanic *Pseudoalteromonas lipolytica* [156]. An unexpected twist is a point mutation in the 5′ untranslated region of the fatty acid biosynthesis *accBC* gene cluster which might activate the Wsp signalling pathway by alteration of the fatty acid composition of the membrane [157].

Novel biofilm types can also arise upon co-operation of different mutants. *P. fluorescens* Pf0-1 reversibly displays two different colony morphology types, mucoid and dry [158]. Mixing of these two colony types triggers a cooperative territory gaining behaviour towards nutrient-rich surfaces which requires the capsular polysaccharide production of the mucoid strain and the high cyclic di-GMP production of the dry isolate. High cyclic di-GMP production (presumably coupled with the production of a biofilm exopolysaccharide) was achieved in the dry colony initially due to gene fusion of the scaffolding gene *wspD* with the methyl-transferase gene *wspC*, a missense mutation in the histidine kinase WspE and with the methyl-accepting chemotaxis gene *wpsA* which presumably leads to a higher frequency of activation of the downstream response regulator, the diguanylate cyclase WspR [159]. Extended sequential mutational analysis still identified the Wsp system as the major initial mutational hub to regulate cyclic di-GMP levels in the mucoid/dry colony morphology switch with subsequent involvement of alternative diguanylate cyclases and gene products associated with changes in cyclic di-GMP levels [159, 160]. In other cases does a mutation that promotes elevated cyclic di-GMP signalling not occur in the cyclic di-GMP turnover proteins itself, but in associated sensory proteins or predicted cyclic di-GMP receptors such as IlvH, a regulatory subunit involved in the first steps of synthesis of branched chain amino acids.

As an example of beneficial species interaction, the arisal of high biofilm formers can support synergistic biomass production [161]. In the *Xanthomonas retroflexus/Paenibacillus amylolyticus* two species biofilm model mutualistic interactions were enhanced by the appearance of mutation in predominantly one distinct diguanylate cyclase gene that promoted higher biofilm formation of *X. retroflexus* with subsequently elevated biomass even for the interacting *P. amylolyticus *.

## Conclusions

The cyclic di-GMP signalling system is one of the most prevalent and adaptable signalling system in bacteria. Variability of cyclic di-GMP signalling has been shown to occur by different mechanisms from promoter switch mutations over single and multiple amino acid substitutions to gene truncations, domain swaps and novel horizontally introduced gene products. While variability has been frequently observed, the evolutionary forces that lead to those mutagenic events equally as their impact on network rewiring and physiological and metabolic consequences are not readily accessible. Although the rule rather than the exception in natural isolates and frequently even in evolutionary microcosm experiments, a mutation in the cyclic di-GMP signalling system is associated with additional mutation(s) on the genome. Thus, natural and cosmos derived mutations are a treasure source not only for investigation of the structural and catalytic impact of often unexpected amino acid substitutions in cyclic di-GMP turnover proteins, but also for the discovery of novel physiological, metabolic and morphological roles of the cyclic di-GMP signalling system.
